# The antimicrobial effect of different vitamin D compounds on *Streptococcus mutans* and their impact on glycosyltransferase expression

**DOI:** 10.1080/20002297.2024.2327758

**Published:** 2024-03-27

**Authors:** Marta Picolo, Abish Stephen, Aylin Baysan

**Affiliations:** a Centre for Oral Bioengineering, Institute of Dentistry, Barts and The London School of Medicine and Dentistry, Queen Mary University of London, London, UK; b Centre for Oral Immunobiology and Regenerative Medicine, Institute of Dentistry, Barts and The London School of Medicine and Dentistry, Queen Mary University of London, London, UK

**Keywords:** Vitamin D, dental caries, streptococcus mutans, glycosyltransferase, cholecalciferol, doxercalciferol

## Abstract

**Background:**

*Streptococcus mutans* is a virulent microorganism associated with dental caries. This *in vitro* study aimed to investigate the antimicrobial effects of Cholecalciferol (D3) and Doxercalciferol (D2), against *S.* *mutans* and on glycosyltransferase gene expression.

**Methods:**

Minimum inhibitory concentration (MIC) and minimum bactericidal concentration (MBC) of D3 and D2 for *S.* *mutans* were determined according to the Clinical Laboratory Standards Institute guidelines. The effect of the compounds on environmental pH in 1% w/v and 5% w/v sucrose broth cultures after 24 hours were assessed colorimetrically. Additionally, their impact on glycosyltransferases gene expression (*GtfB, GtfC, GtfD*) in 5% w/v sucrose culture was evaluated using quantitative real-time PCR.

**Results:**

The MBCs of D3 and D2 were 83 µg/ml and 166 µg/ml respectively. Both compounds were effective in preventing the local pH drop <5.5 at ≥166 µg/ml in sucrose supplemented cultures. However, the compounds did not inhibit pH drop at MIC values. Notably, D2 upregulated *GtfD* expression significantly (*p* < 0.05) and downregulated *GtfB* and *GtfC*.

**Conclusion:**

Vitamin D2 and D3 inhibited *S. mutans* mediated pH drop in sucrose supplemented cultures and altered glycosyltransferase expression, suggesting potential therapeutic roles in dental caries prevention. Further research is needed to assess their full impact on *S. mutans* survival under environmental stresses.

## Introduction

Dental caries is a multifactorial and infectious disease [1] estimated to affect more than two billion individuals worldwide [2]. The onset of dental carious lesions depends on the action of various microorganisms [3,4]

*Streptococcus mutans* is one of the main bacterial species involved in dental caries initiation [1,5,6. *S. mutans* is recognised as a biofilm promoter by the ability to adhere to the tooth structure and allowing the binding of other microorganisms by secreting extracellular glucans. In addition, this gram-positive bacterium, which is acidogenic and aciduric, can promote a decrease in local pH resulting in hard tissue demineralisation. The resistance of *S. mutans* to ecological shifts allows the organism to outcompete other oral commensal micro-organisms [7].

*S*. *mutans* synthesises three main types of glycosyltransferases; *GtfB, GtfC, and GtfD,* which are responsible for the bacterial adhesion in sucrose environments [8]. *GtfB* and *GtfC* synthesise mostly cell surface water-insoluble glucans, with α-1,3-glycosidic linkages [9,10]). *GtfD* generates water-soluble glucans, composed by α1,6-glucosidic linkages (Hanada and Kuramitso, 1999). Glucan synthesis by *GtfB* and *GtfC* allows for the formation of a matrix that can facilitate adherence to the tooth surface and adsorption of other bacterial cells by enhancing the establishment of dental biofilm [11–13]. Therefore, the Gtf activity inhibition and polysaccharide synthesis may reduce the virulence effect of cariogenic biofilms on tooth, which in turn could be considered as a preventative strategy for dental caries.

In addition, the biofilm bacteria can generate organic acids from their metabolisms. Their release into the extracellular media lowers the local pH and leads to hydroxyapatite dissolution [14]. The acidic subproducts released, dissociate as anions, and react with protons, contributing to the pH drop. In turn, the protons diffuse into the microorganism’s cytoplasm, promoting its acidification. The cytoplasmic pH drop is unfavourable to bacteria as they possess acid-sensitive enzymes that may lose their function and damage DNA [15].

*S*. *mutans* develops effective mechanisms for the cytoplasmic pH to be maintained and to be protected from metabolism breakdown [16]. When exposed to lower pH environments, such as under pH 4.0, *S. mutans* protects its glycolytic enzymes by carrying the protons across the cell membrane through F-ATPase [17]. The membrane of this bacterium is composed of water-insoluble glucans (namely α-1,3-linked glucans) that entrap protons [18] and allow adaptation to acidic environments. Research into transcriptomic analysis reported a positive association between sucrose-dependent glucans biosynthesis and acid tolerance [19].

Small molecules such as molecular weight <1 kDa, both natural and synthetic [20] are shown as promising alternatives for preventing dental caries, as these molecules present enhanced cell permeability and stability, low toxicity, and satisfactory costs [21,22]. A broad range of these agents have been identified as effective against the virulence effect of *S. mutans* [23]. In this respect, [24] assessed a total of 853 FDA-approved drugs, identifying 126 antimicrobial candidates including Vitamin D compounds, i.e. Alfacalcidol, Calcitriol, and Doxercalciferol against *S. mutans*.

The National Diet and Nutritional Survey in the UK estimated that one in six adults presents with deficient serum levels of Vit D, which is believed to be related to the substandard skin exposure to sunlight especially during the Autumn and Winter [25]. The World Health Organisation also reported that dental caries is in the top five of most dispendious chronic diseases to treat [26]. Although the mechanism is still unclear, current evidence underlines the possible association between low vitamin D levels and high prevalence of dental caries in all age groups [27–30].

Alfacalcidol, Calcitriol, and Doxercalciferol could inhibit the growth of *S. mutans* and biofilm formation. In addition, the synergic activity of the latter and Bacitracin against this micro-organism was also acknowledged [24]. Recently, Cholecalciferol (D3) was shown to be effective against *S. mutans* and *Streptococcus sobrinus*. Scanning Electron Microscope (SEM) analysis reported that both microorganisms presented morphological changes following the application of D3 [31]. Vitamin D compounds may be promising for the prevention of carious lesions. However, there is limited evidence related to Vitamin D in the management of dental caries. Therefore, the aim of this study was to investigate the mechanism(s) by which Cholecalciferol and Doxercalciferol affect *S. mutans* by exploring the effect on the microorganism’s cariogenic potential.

## Materials and methods

### Compound preparation

Eight milligrams of Doxercalciferol (ApexBio, Houston, Texas No: B2091) and Cholecalciferol (Sigma Aldrich, Germany No: 47763) were measured and added to a sterile 5 ml glass flask. Following this, 900 µl of 100% ethanol and 100 µl of distilled water were added. The solutions were vortexed, until the full dissolution of these compounds and stored at −20°C for future use.

### Culture of Streptococcus mutans

*Streptococcus mutans* (Strain, NCTC 10,449) was obtained from stock (−80°C) and maintained aerobically in agar plates – Columbia Agar (Oxoid, Hampshire, UK) with 5% defibrinated horse blood at 37°C and 5% CO_2_.

### Minimum inhibitory concentration (MIC) and minimum bactericidal concentration (MBC)

Colonies of *S. mutans* were retrieved from agar plates and inoculated in trypticase soy broth (TSB; Oxoid, Hampshire, UK) with 1% glucose and incubated overnight at 37°C and 5% CO_2_. The bacterial culture was centrifuged (Thermo, Sorvall Legend T+, Centrifuge, USA) at 1600*g* for 5 min, and the supernatant discarded. Bacterial cell suspensions of 0.2 OD_600_ were prepared in TSB. A sterile 96-well plate (Thermo, Hampshire, UK) was used to execute the microdilution protocol in a total volume of 200 µL [32,33]. Each compound was tested as doubling dilution series, with 90% ethanol and triplicate positive controls included (TSB and *S mutans*). Using a plate reader (CLARIOstar, BMG LABTECH, UK), the OD600 of the solutions were read at baseline and for a period of 24 h at the endpoint (overnight incubation at 37°C and 5% CO_2_).

To assess the MBC value for both compounds, decimal dilutions were carried out from the MIC solutions. A volume of 50 µl was then inoculated in agar plates, then incubated for 24 h at 37°C and 5% CO_2_. Subsequently, the colonies were counted. The MIC and MBC experiments were repeated thrice.

### pH assay

The pH assay was performed following the microdilutions protocol [32] both for 5% and 1% sucrose TSB, with bromocresol used as a pH indicator. The absorbance values were then read at baseline and 24 h after the incubation (5% CO_2_). A standard curve for the pH ranges from 4.0 to 7.0 was prepared with bromocresol. The pH assay was repeated thrice.

### *Expression of glycosyltransferases* (gtfs)

Three different concentration solutions (6 mg/ml, 0.5 mg/ml, and 0.065 mg/ml) were prepared for both Cholecalciferol (D3) and Doxercalciferol (D2). The lowest concentration prepared (0.065 mg/ml) stands below the MIC as determined in the MIC assay. In addition, the 0.5 mg/ml solution aimed to stand centrally between 0.333 mg/ml and 0.666 mg/ml as tested in the MIC assay.

*S*. *mutans* was exposed to different concentrations of both test compounds and no-treatment (control) group to investigate the effect of Vit D in the expression of genes related to the cariogenicity of this micro-organism. *S. mutans* growing in 5% (w/v) sucrose TSB was inoculated into fresh sucrose-TSB containing Doxercalciferol and Cholecalciferol at three different concentrations (6 mg/ml, 0.5 mg/ml, and 0.065 mg/ml). The cultures were incubated for 24 h at 37°C and 5% CO_2_ before pelleting at >10,000 g and the RNA from the harvested cells was extracted using the RNeasy kit (Qiagen, Germany), according to the manufacturer’s instructions.

RNA quantity was then measured using a spectrophotometer (DeNovix, USA) and equimolar quantities of cDNA were prepared using the Quantiscript RT kit (Qiagen). qPCR assays were performed using SYBR Green I (Roche), in 10uL total volume and 0.4 µM of forward and reverse primers *per* well. The samples were transferred into a thermocycler (Roche, Switzerland) and exposed to an activation phase (50°C), denaturation (95°C). A total of 40 amplification cycles of 60°C (1 min) and 95°C (3 s). Finally, the melting curve and cooling stage were programmed.

The primers used are presented in Table 1. A housekeeping gene (16S rRNA) was used as a normaliser for the gene expression data due to its stability under a variety of environmental conditions.

**Table 1.** Validated primer sequences (forward and reverse) used in this study for the evaluation of each glycosyltransferase B, C, and D in *S. mutans.*

Primer	Forward	Reverse
*GtfB*_[34]_	5’-AGCAATGCAGCCAATCTACAAAT-3’	5’-ACGAACTTTGCCGTTATTGTCA-3’
*GtfC*_[34]_	5’-GGTTTAACGTCAAAATTAGCTGTATTAGC-3’	5’-CTCAACCAACCGCCACTGTT-3’
*GtfD*_[34]_	5’-ACAGCAGACAGCAGCCAAGA-3’	5’-ACTGGGTTTGCTGCGTTTG-3’
*16S*	5’-CCTACGGGAGGCAGCAG3’	5’-TTACCGCGGCTGCTGG3’

## Results

### Minimum inhibitory concentration (MIC) and minimum bactericidal concentration (MBC)

**Figure 1.**
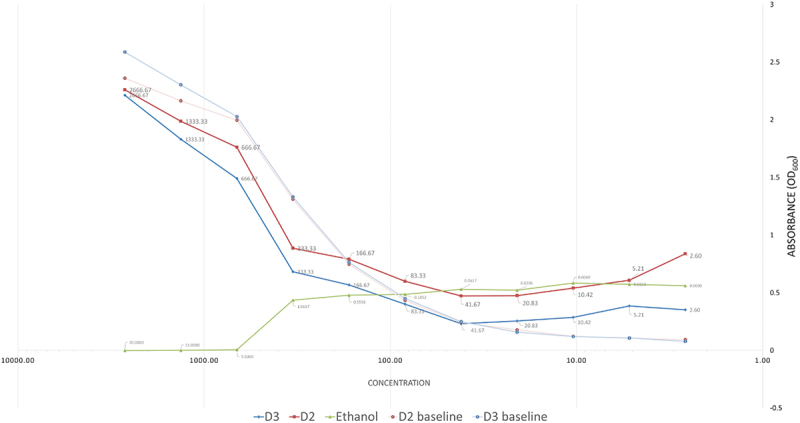
Graph representing measured baseline and endpoint absorbance values for doxercalciferol (D2), Cholecalciferol (D3) and ethanol in the broth microdilution assay, with the x-axis shown in log-scale. The compound (µg/ml) and ethanol (v/v) concentrations are indicated in the data labels.

The reported MIC was between 41–83 µg/ml and 83–166 µg/ml for Doxercalciferol (D2) and Cholecalciferol (D3) respectively (Figure 1). The MBCs of both compounds obtained were 83 µg/ml for Cholecalciferol and 166 µg/ml for Doxercalciferol, since no growth was observed in the viable count experiments. The ethanol concentration used for dissolving the compounds was subjected to a series of doubling dilutions, demonstrating that bacterial inhibition was not observed beyond the 4th dilution (Figure 1), corresponding to an ethanol concentration of 1.67% v/v i.e. the MIC observed for the compounds was at 83 µg/ml for Cholecalciferol, at which the ethanol concentration was only 0.18% v/v. The bactericidal effect observed was therefore attributable to the compounds rather than the ethanol content in the dilutions.

### pH assay

**Figure 2.**
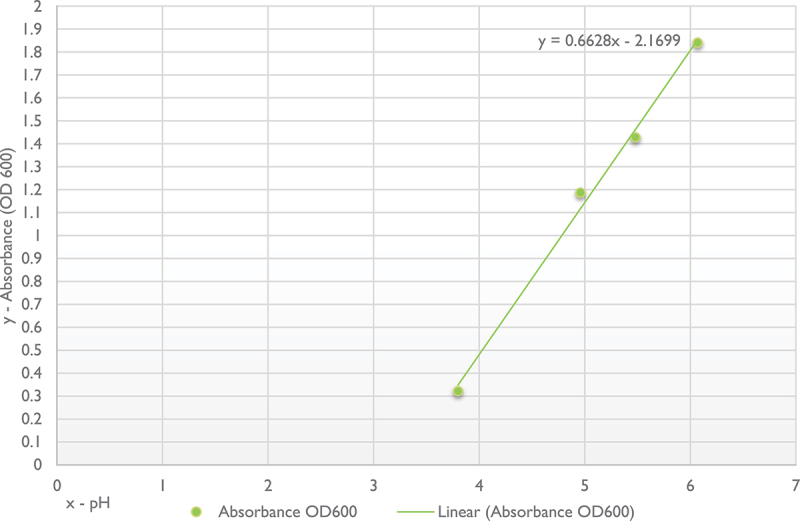
Relationship between pH and absorbance values.

A linear regression correlating the pH and absorbance value was obtained (Figure 2). The correlation was observed at the pH intervals between 3.8 and 6.

### pH assay − 1% sucrose broth

**Figure 3.**
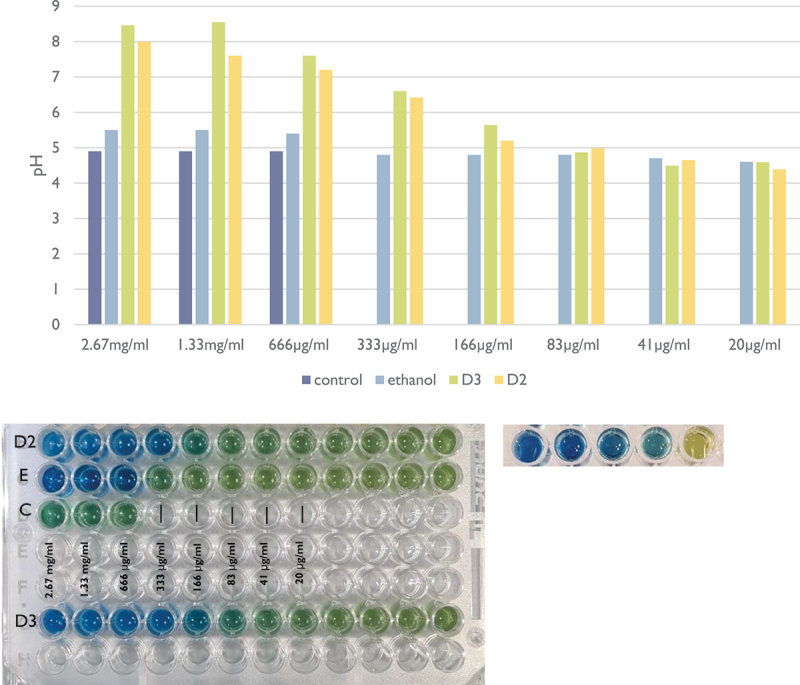
pH assay in 1% sucrose medium. D3: Doxercalciferol; D2: Cholecalciferol; E: Ethanol; C: control (TSB 1% sucrose and bacteria). The graph corresponding to the values of pH obtained by the linear regression can be seen above (y-axis = pH; x-axis = vitamin D concentration).

In the pH assay with 1% sucrose, the presence of both Cholecalciferol (D3) and Doxercalciferol (D2) inhibited the drop in local pH at concentrations of ≥83 µg/ml in comparison to the control and solvent (ethanol) groups. According to the colour panel, the change in pH indicator was evident at 83 µg/ml and at 166 µg/ml for D3 and D2, respectively. The absorbance values of the separate set of experiments were read and presented in Figure 3.

### pH assay − 5% sucrose broth

**Figure 4.**
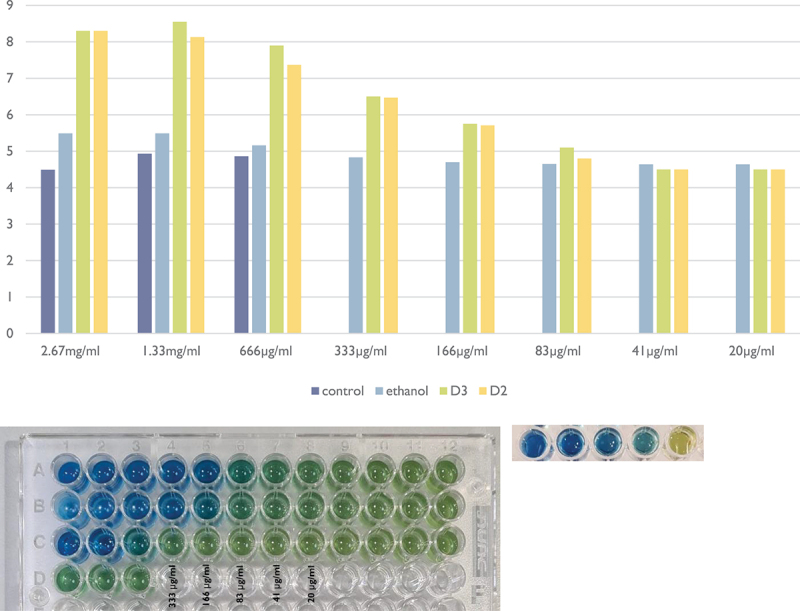
pH assay in 5% sucrose medium. D3: Doxercalciferol; D2: Cholecalciferol; E: Ethanol; C: control (TSB 5% sucrose and bacteria). The graph corresponding to the values of pH obtained by the linear regression can be seen above (y-axis = pH; x-axis = vitamin D concentration).

Figure 4 demonstrates the pH assay in 5% sucrose medium. The presence of both Cholecalciferol (D3) and Doxercalciferol (D2) inhibited the drop in local pH at concentrations of ≥83 µg/ml in comparison to the control and solvent (ethanol) groups. According to the colour panel, the change in pH indicator was evident at 83 µg/ml for both D2 and D3.

At 166 µg/ml concentration, the pH was 5.76 for D3 whilst the value dropped to 5.12 at 83 µg/ml. Similarly, D2 demonstrated a pH value of 5.72 at 166 µg/ml. Furthermore, the pH for D2 dropped to 4.82 at 83 µg/ml.

### Expression of glycosyltransferases

The results from the RT-qPCR were evaluated after calculation of ΔCt to assess the differences in gene expressions of *GtfB, GtfC*, and *GtfD* following the applications of different compound concentrations (6 mg/ml, 0.5 mg/ml, and 0.065 mg/ml) in comparison to the negative control group (no treatment).

#### GtfB

**Figure 5.**
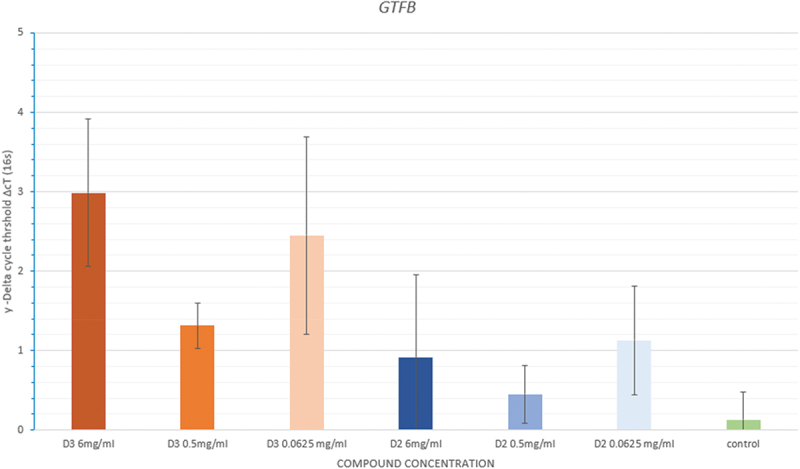
Mean in delta cycle threshold (ΔcT) of the different concentrations of cholecalciferol (D3), Doxercalciferol (D2) and negative control group for *GTFB*.

The delta cycle threshold (ΔCt) values were calculated relative to a housekeeping gene (16S). Figure 5. shows the differences in the delta cycle threshold. There was evidence of a similar pattern between the two compounds. However, samples treated with D3 showed reduced expression in comparison to the D2, relatively to the mean ΔCt of the negative control group. However, the mean ΔcT values either between D3 or D2 and negative control groups were statistically insignificant (*p* = 0.081; *p* = 0.652 respectively).

There was downregulation of the gene at all the concentrations tested for both compounds as the ΔcT exceeds the negative control group. In addition, a similar pattern was observed in both compounds, where the downregulation appears to be higher at 0.5 mg/ml.

#### GtfC

The mean ΔcT either between D3 or D2 group and negative control was statistically insignificant (*p =* 0.149; *p =* 0.891 respectively).

**Figure 6.**
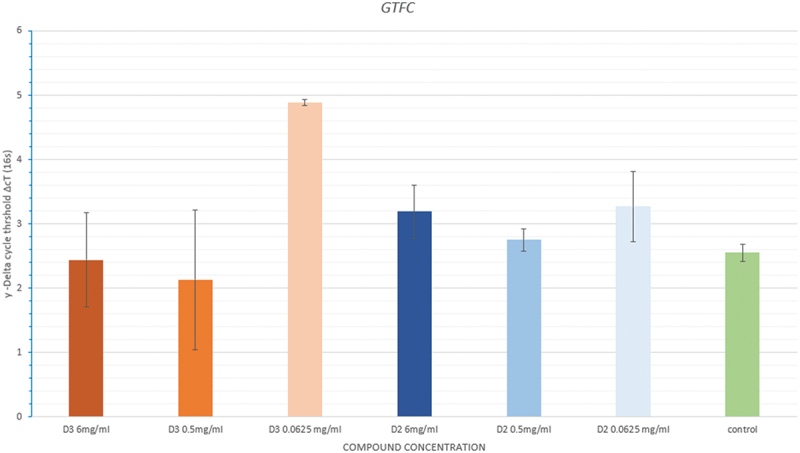
Mean in delta cycle threshold (ΔcT) of the different concentrations of cholecalciferol (D3), Doxercalciferol (D2) and negative control groups.

The results showed a similar pattern between both compounds. However, there was a difference between the samples treated with the lowest concentration of both D3 and D2, when compared to the ΔCt of the control, as D3 (0.0625 mg/ml) showed to be more effective (Figure 6).

#### GtfD

**Figure 7.**
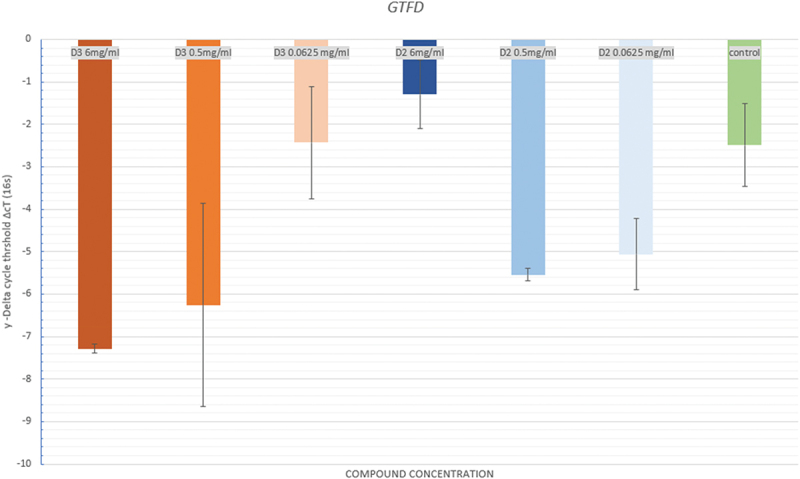
Mean in delta cycle threshold (ΔcT) of the different concentrations of doxercalciferol (D2), Cholecalciferol (D3), and negative control groups for GtfD.

Figure 7. demonstrates the mean ΔcT between the D3 concentrations and negative control was statistically insignificant (*p =* 0.125). However, the mean ΔcT between D2 groups and negative control was statistically significant (*p =* 0.013)

There was overexpression of GtfD as the ΔcT was lower in comparison to the negative control group. To the normalisation gene, the negative control was −2,49. For D3 (6 mg/ml and 0.5 mg/ml), the ΔcT was −7.285 and 6.25. In addition, for D2 (0.5 mg/ml and 0.0625 mg/ml), the ΔcT values were −5.5 and −5.0, respectively. Therefore, the expression occurred quickly for the treated samples. The latter failed to occur for D3 at the lowest concentration tested nor D2 at the high concentration tested as the ΔcT values were close to the x-axis relatively to the control group.

## Discussion

This laboratory-based study aimed to evaluate the effect of Doxercalciferol (D2) and Cholecalciferol (D3) in inhibiting the cariogenicity of *S. mutans*. This study was the first to evaluate the minimum bactericidal concentration (MBC) values on *S. mutans* following the application of Doxercalciferol. The MBC values obtained for both compounds in this study were 166 µg/ml and 83 µg/ml for Doxercalciferol and Cholecalciferol respectively. This contrasts with Almoudi et al. [31], who reported the MBC for Cholecalciferol of 500 µg/ml. Several factors such as water solubility and polarity, which are influenced by preparation methods, may impact the Minimum Inhibitory Concentration (MIC) readings [35,36]). Notably, both Doxercalciferol and Cholecalciferol are hydrophobic and available in powder form, necessitating the use of solvents such as ethanol or dimethyl sulfoxide (DMSO) for dissolution [37,38]. DMSO commonly employed for diluting hydrophobic agents in biological assays is known to have a possible detrimental effect on the cell membrane, structure, and properties even at low concentrations [39]. The use of DMSO may also compromise the bacterial membrane and inhibit cell growth [40]. Contrarily, ethanol shows toxicity against bacteria at high concentrations (substantial %v/v) [41,42]. The addition of water to diminish the solvent toxicity results in high turbidity solutions, which can also compromise the reading of absorbance values. Adding water to the solution may also lead to solvent evaporation, which is fast for ethanol in comparison to water [43,44].

In addition, microbial resistance levels are not static and may fluctuate due to environmental conditions and the state of the micro-organism [45,46]. The MIC for *S. mutans* might significantly be affected by its different strains, meaning that for the same bacteria, the resistance level may increase or decrease depending on the strain tested [47].

The ability of *S. mutans* to produce acid in the presence of Vitamin D has not been extensively investigated. The critical pH value for enamel is 5.5 [48]. Results in this current study indicated that both compounds were able to maintain a pH above 5.5, until certain concentrations, both in 1% and 5% sucrose media. With regard to 1% sucrose assay, Cholecalciferol (D2) at 166 µg/ml and Doxercalciferol (D3) at 333 µg/ml showed the optimum results and inhibited the pH reduction below the critical value. However, for the 5% sucrose assay, both compounds inhibited the pH reduction at 166 µg/ml and maintained a pH of 6 or higher at 333 µg/ml concentration.

These concentrations exceed the MBC for both compounds, which can be beneficial against the resistance for *S. mutans* when exposed to a 5% sucrose medium. It was previously reported that 3% sucrose environments increase the resistance of *S. mutans* to certain inhibitors [49–51]. Both compounds were effective in inhibiting local pH drop at MIC concentrations in both 1% and 5% sucrose broths when compared with the control and ethanol groups, namely at concentrations ≥83 µg/ml. Al-Jubori et al. [52], recently evaluated the remineralising potential of D3 gel application (1000UI) on demineralised enamel and reported significant remineralisation potential on early enamel lesions which was justified by the increase in mineral content and surface microhardness. It can be speculated that Vitamin D and fluoride could potentially improve remineralisation under acidic conditions. Both fluoride [53] and Vitamin D were reported to be effective against *S. mutans* [31,36,54]. Almoudi et al. [31], observed a substantial change in morphology, i.e. intracellular material leakage, cell distortion and extracellular membrane damage/rupture after exposing *S. mutans* to Vitamin D3.

The bacterial cell wall, crucial for survival and colonisation, presents a significant barrier for antimicrobial agents, especially in gram-positive bacteria, such as *S. mutans*, which possess a thick peptidoglycan layer [55–57]. The synergic use of fluoride and Vitamin D compounds could improve remineralisation by inhibiting the numbers and colonisation of *S. mutans*.

The reported synergistic effect of Bacitracin and Doxercalciferol against *S. mutans*, potentiating the inhibition was at MIC of 4 µg/ml, whereas bacitracin alone was >128 µg/ml [36]. These authors indicated that one of the mechanisms of Vitamin D2 might be associated to a downregulation of efflux pump systems. Some strains of *S. mutans* encode for an antiporter responsible for expelling fluoride from the cytoplasm and this pump could be disrupted by the action of Vitamin D [58].

This study also represented the first evaluation of gene expression related to biofilm formation by *S. mutans*. The results were statistically insignificant for the ΔCt values between the test groups, *GtfB* and *GtfC*. However, the mean difference in ΔCt between groups was significant for *GtfD* and Doxercalciferol only (*p* = 0.013). This was not the case for Cholecalciferol for this specific gene (*p* = 0.125). Doxercalciferol was shown to inhibit biofilm formation in *S. mutans* at a concentration of 128 µg/ml [36]. In addition, severe membrane alterations and bacterium-to-bacterium contact were reported following the application of cholecalciferol (250 µg/ml) [31]. In this current study, the delta cycle threshold calculations at 0.5 µg/ml and 6.25 ng/ml for Doxercalciferol (D2) suggested that the expression of *GtfB* and *GtfC* occurred at 60% and 55%. Furthermore, Cholecalciferol (D3) was effective in downregulating both genes, occurring at 19.8% and 20.8% when compared to the non-treatment group. Almoudi et al. [31], observed alterations in the bacterial cell membrane and cell-to-cell relationship after the Vitamin D3 exposure. The current results showed an increase in the expression of *GtfD* and a decrease in *GtfB* and *GtfC*. Therefore, a great downregulation after Cholecalciferol (D3) exposure was reported, in comparison to the Doxercalciferol (D2), which can suggest the reliability of the reported MIC intervals in comparison to the studies by Almoudi et al. [31,36].

The three studied *Gtfs* are genetically distinct for *S. mutans* and play different detrimental roles in the virulence of dental plaque [59]. *GtfB* synthesises insoluble glucans (α-1.3-linkage), whilst GtfC produces both soluble and insoluble glucans and *GtfD* is responsible for the production of mostly soluble glucans [9,60]. Importantly, *GtfB* can penetrate to enamel (at lower rates than *GtfC*) and adhere to other bacterial species, such as *Actinomyces, Lactobacillus casei* and *S. mutans* [61]. *GtfC* adsorbs the enamel pellicle, and *GtfD* increases the production of metabolisable polysaccharides [59]. Furthermore, the latter serves as a primer for *GtfB* expression and is a source of metabolism for other biofilm bacterial species [62].

The current findings indicated the overexpression of *GtfD* (*p* = 0.013) could relate to the attempt of *S. mutans* to produce soluble glucans that could aid in *GtfB* expression. Therefore, it can be speculated that the under expression of *GtfB* was observed. It was previously reported that other biofilm micro-organisms produce metabolites that act as primers for *GtfB* [11,63]. Since overexpressing *GtfD* failed to result in *GtfB* synthesis, *S. mutans* potentially attempted to provide neighbouring bacteria with *GtfD*, to receive subproducts that would allow to enhance the expression of *GtfB*.

The inability of *S. mutans* to fully express *GtfB* reduces its cariogenicity. This gene promotes the synthesis of α-1,3-linked glucans, which compose the plasma membrane and potentiate the adaptation of *S. mutans* to low pH environments. These glucans form a tight membrane that entraps protons and inhibits fluctuations of cytoplasmic pH [18]. In this current study, the pH assay failed to show any improvements in the environmental pH after the Vitamin D application (at MIC value), however, it can be speculated that the bacteria’s ability to tolerate acidic pH mediums could have been compromised due to the under expression of *GtfB*. Both the pH assay and gene expression were carried in sucrose-rich broths, leading to speculation that aciduricity could be weakened. *GtfB* expression is upregulated when *S. mutans* is exposed to a sucrose-rich medium [64,65]. In this study, the *GtfB* expression was downregulated in the presence of both compounds. It should be noted that *GtfC* and *GtfB* are highly homologous (75% similar in amino-acid sequence) and ruled by the same regulatory mechanisms. However, *GtfD* is up to 50% similar to *B* and *C* and is ruled by distinct mechanisms [66]. Furthermore, *GtfD* is located upstream of the locus that encapsulates *GtfB* and C, demonstrating an independent promoter [67]. Although *GtfD* can bind to hydroxyapatite, the lower binding sites with this gene were reported when compared to *GtfB* and *GtfC*. In addition, the inability to promote bacterial colonisation as efficiently as *GtfB* was previously indicated [63].

The ability to form biofilm and adhere to enamel may be compromised in the presence of 5% sucrose and low Vitamin D compound concentrations (65 µg/ml). The current study followed the model proposed by Rölla et al. [68] regarding the *Gtf*-glucan-mediated biofilm formation. However, further evidence is required to evaluate the effects of these agents in the expression of virulent genes of *S. mutans*. A decrease in the expression of *Gtfs* in the presence of low concentrations of Vitamin D suggested a possible unique preventive strategy for dental caries. Although this study failed to find statistical significance in cycle threshold values, it could potentially lead to further evidence on the effects of Doxercalciferol (D2) and Cholecalciferol (D3) for the virulence factors of *S. mutans*.

It should also be noted that the production of *Gtf*-like enzymes is not exclusive to *S. mutans*. *Streptococcus sobrinus, Actinomyces*, and *Lactobacilli* synthesise similar ones [69]. Therefore, it can be speculated that these compounds might be effective against other cariogenic bacteria.

The recommended adult dosage of Vitamin D supplements is 400 IU (10 µg) *per* day (NHS [70]), which could be applied in a gel form or chewable table to potentially promote remineralisation and decrease virulent biofilm formation by *S. mutans*.

In this respect, the British National Diet and Nutrition Survey concluded that one in six British individuals are Vitamin D deficient [25], and by 2023 the percentage of individuals taking vitamin D supplements decreased [71]. Vitamin D supplementation may increase salivary flow rates and further increase the anti-caries action of saliva. The deficiency in Vitamin D was previously associated with diminished salivary flow rates, as well as reduced parotid gland function [72]. Accordingly, D3 supplementation increased salivary flow rates in rats [73]. He et al. [74], demonstrated that after supplementation with D3, salivary flow rates in healthy male athletes (*n* = 39) increased with time. Additionally, the authors found Vitamin D receptors in all salivary glands, suggesting that this vitamin could play a role in controlling salivary secretion [74]. The current study evaluated the antimicrobial and anticariogenic effects of different concentrations of two Vitamin D compounds 24 h post application. Although the usage of chewable or gel form of Vitamin D would have a periodic effect, the application of both D3 or D2 could act as preventive strategies for biofilm formation and dental carious lesions, as well as contribute to overall general health.

The current study is presented with few limitations. This was a laboratory-based study, therefore the methodology does not mimic the oral cavity). The study also focused on one cariogenic micro-organism alone and the effect of Vitamin D compounds on different micro-organisms need to be investigated. The colony forming units were counted manually, which is prone to potential error(s) [75]. Further studies are required to investigate the effect of Vitamin D agents on different cariogenic micro-organisms and their impact on gene expressions.

## Conclusion

Within the limitations of this laboratory-based study, both Vitamin D2 and D3 agents presented antimicrobial effects against *S. mutans*. Both agents were effective in inhibiting the local pH drop, both in 1% and 5% sucrose broths at MIC concentrations when compared to the control and ethanol groups. According to the RT-qPCR results, the expressions of *GtfB* and *GtfC* were downregulated, whilst an overexpression of *GtfD* was noted. *GtfB* forms the extracellular glucan matrix that enables endurance of the cells in low pH environments. Therefore, the under expression might compromise the survival of *S. mutans* in critical environmental changes.

While these findings suggest that the standard dosage of Vitamin D (400 IU) could potentially combat the formation of virulent biofilms by *S. mutans*, further in-depth and randomised clinical trials are necessary to validate these observations. This vitamin could then be used to improve individual caries experience, oral health, and ultimately overall health.
